# Carbon Dots-TiO_2_ Decorated with Ag Nanoparticles for Efficient Photocatalytic and Antiviral Applications †

**DOI:** 10.3390/ma19102084

**Published:** 2026-05-15

**Authors:** Alexandra Karagianni, Adamantia Zourou, Aekkachai Tuekprakhon, Afroditi Ntziouni, Anna-Maria Tavlaridi, Ioanna Kitsou, Dimitra Katerinopoulou, Aspasia Stoumpidi, Georgios Kiriakidis, Zania Stamataki, Konstantinos V. Kordatos

**Affiliations:** 1 School of Chemical Engineering, National Technical University of Athens (NTUA), 9 Iroon Polytechniou St., Zografou, 15780 Athens, Greece; alexakar1510@outlook.com (A.K.); afro.ntzio@gmail.com (A.N.); ananmartab@gmail.com (A.-M.T.); 2 Centre of Liver and Gastrointestinal for Research, Department of immunology and Immunotherapy, School of Infection, Inflammation and Immunology, College of Medicine and Health, University of Birmingham, Birmingham B15 2TT, UK; a.tuekprakhon@bham.ac.uk (A.T.); z.stamataki@bham.ac.uk (Z.S.); 3 School of Mining & Metallurgical Engineering, National Technical University of Athens, 9 Iroon Polytechniou St., Zografou, 15780 Athens, Greece; kitioanna@metal.ntua.gr; 4 Photo-Catalytic Nano Materials (PCN) Materials, Craftsmen Industrial Park of Heraklion, 5 Knossou St., Anopolis, 70008 Heraklion, Greece; dkaterin@pcnmaterials.com (D.K.); aspa@pcnmaterials.com (A.S.); kiriakid@pcnmaterials.com (G.K.)

**Keywords:** titanium dioxide (TiO_2_), carbon dots, silver (Ag) nanoparticles, photocatalysis, pollutants degradation, antiviral activity, SARS-CoV-2 inactivation

## Abstract

The modern world is confronting critical environmental and biomedical challenges, underscoring the urgent need for the development of multifunctional materials—an inherently interdisciplinary field, bridging materials science and engineering, environmental science and biomedicine. Titanium dioxide (TiO_2_) is widely recognized for its photocatalytic and antiviral properties, enabling the degradation of pollutants and mitigation of viral contamination under solar irradiation. Nevertheless, it exhibits certain limitations, such as wide band gap and high recombination rate of photogenerated electron–hole pairs. To address these limitations, TiO_2_ prepared by a co-precipitation method was modified with N-Doped Carbon Dots (N-CDs) via a hydrothermal treatment, which extend light absorption into the visible region and enhance charge separation. Further functionalization with silver nanoparticles (Ag NPs)—well known for their antimicrobial properties—via a simple thermal process under ambient conditions, introduced additional reactive oxygen species generation, creating a synergistic effect. The as-prepared TiO_2_, TiO_2_/N-CDs and TiO_2_/N-CDs/Ag samples were characterized via several techniques, such as XRD, micro-Raman, FT-IR, TEM and UV-Vis. In addition, their photocatalytic and antiviral activity against methylene blue (MB) and nitrogen oxide (NO_x_) pollutants, as well as SARS-CoV-2, was evaluated. Based on the results of liquid-phase photocatalysis, TiO_2_, TiO_2_/N-CDs and TiO_2_/N-CDs/Ag presented a degradation efficiency of 78%, 85% and 95%, respectively, whereas different trends were observed under gaseous-phase conditions. The TiO_2_/N-CDs/Ag hybrid material demonstrated superior antiviral activity against SARS-CoV-2 (IC_50_: 1.24 ± 0.34 g/L), compared to both TiO_2_ (IC_50_: 1.78 ± 0.30 g/L) and TiO_2_/N-CDs (IC_50_: >2.5 g/L), highlighting its potential as an effective multifunctional material. Finally, TiO_2_/N-CDs/Ag was incorporated onto a paper substrate, demonstrating antiviral activity, showing promising scalability for application across a wide range of future substrates. To the best of our knowledge, this is the first study presenting TiO_2_/N-CDs/Ag with dual photocatalytic and antiviral activity.

## 1. Introduction

Global concerns associated with water and air pollution, as well as recurring viral outbreaks, highlight the urgent need for the fabrication of materials with dual action on environmental and public health protection. Water pollution, in particular, arises from a combination of human-driven and natural factors, such as industrial discharge, agricultural activities, urbanization, climate change, etc. All these factors release persistent heavy metals, dyes, pesticides, organic farm waste, pharmaceuticals, polycyclic aromatic hydrocarbons and pathogens into aquatic systems. In many developing regions, these challenges are acute and lead to the degradation of water quality, posing severe threats to ecosystem stability, human health and sustainable development [1]. Among the various types of organic pollutants, synthetic dyes constitute one of the most hazardous contaminants, although they are extensively used in several industries, including pharmaceutical, food, leather, and paper manufacturing. Their widespread release into ecosystems raises serious environmental concerns due to their high chemical stability, toxicity and resistance to conventional wastewater treatment processes. Photocatalysis has emerged as one of the most efficient and sustainable approaches to degrade synthetic dyes and transform them into non-toxic components with the aid of light. Photocatalytic degradation of methylene blue (MB) represents one of the most widely explored model reactions for the assessment of photocatalytic materials. MB is frequently used as a common pollutant due to its intense and well-defined absorbance in the UV-Vis region. In addition, MB possesses increased aqueous solubility. These features allow the monitoring of degradation kinetics and facilitate the conduction of reproducible studies. Beyond water pollution, air pollution stems from both industrial and everyday human activity, leading to the emission of different types of gases, including carbon oxides (CO, CO_2_), nitrogen oxides (NO_x_), sulfur dioxide (SO_2_), volatile organic compounds (VOCs), etc. The release of these hazardous gases threatens human health, by inducing respiratory diseases, cardiovascular diseases or even cancer [2,3]. In addition to the chemical hazards, exposure to the respiratory pathogens is inevitable. The outbreak of the COVID-19 pandemic clearly demonstrated the implementation of sprint technologies to rapidly detect and handle emerging viral threats, whereas the limitations of conventional antiviral drugs were also addressed [4,5]. Therefore, as worldwide environmental and biological concerns continue to escalate, the demand for materials with enhanced and versatile functionalities has grown substantially. This need has fueled intensive research efforts towards the development of multifunctional nanostructures, a rapidly expanding group of nanoscale systems that combine physical, chemical, optical, magnetic and biological characteristics, enabling their utilization across a wide range of fields. The fabrication of such nanosystems mainly involve approaches such as the development of hybrid or composite materials and surface functionalization [6].

Hybrid materials have garnered considerable attention among the scientific community due to their ability to integrate the advantageous properties of different materials into a single multifunctional system. The synergistic effects among the components result in enhanced physicochemical properties, surpassing those of conventional materials such as polymers, metals and ceramics, opening novel pathways for the design of efficient, sustainable and application-oriented technologies [7]. A basic search on Scopus using the phrase “hybrid AND materials” generates a record of more than 140.000 papers published during the past decade. Among them, titanium dioxide (TiO_2_)-based hybrid materials constitute a particularly significant category due to their pivotal role in diverse fields, including environmental remediation [8,9], energy conversion [10] and storage [11], as well as biomedical applications [12,13]. The unique physicochemical characteristics of TiO_2_, including its photocatalytic activity, high chemical stability, eco-friendliness, and tunable electronic structure, combined with its low cost, make it an ideal component for the development of advanced hybrid systems with enhanced properties [14,15,16,17]. Beyond these attractive features, research interest has also been aroused towards the potential antiviral action of TiO_2_. The antiviral performance of TiO_2_ primarily relies on the oxidation of organic molecules, including surface proteins of viruses upon UV irradiation, thereby facilitating effective virus elimination [18,19,20,21]. Despite its promising potential, pure TiO_2_ exhibits strong photocatalytic and antiviral performance only upon UV light irradiation, which is only a small fraction (∼5%) of the solar spectrum. This limitation restricts its effectiveness in the degradation of environmental pollutants and the inactivation of viruses, especially in cloudy conditions or indoor environments. Furthermore, TiO_2_ suffers from low quantum yield due to the rapid recombination of the photogenerated electron holes (e^−^-h^+^). Thus, it is of the utmost importance to fully unlock the photocatalytic activity of TiO_2_ by not only expanding its light-response range, but also preventing the charge carrier recombination. Under this point of view, several strategies have been proposed, including its combination with carbon-based nanomaterials, or/and metal nanoparticles (NPs) for the fabrication of TiO_2_-based hybrid materials with enhanced photocatalytic and antiviral properties [20].

Carbon dots (CDs), zero-dimensional (0-D) nanoparticles, have emerged as highly attractive candidates for the modification of TiO_2_. More specifically, CDs belong to the large family of carbon-based nanomaterials consisting of an either crystalline or amorphous carbon core and an abundance of surface functional groups, including carboxyl, hydroxyl, or amino groups, depending on the precursors. In recent years, they have attracted considerable research attention due to their small size (<10 nm), superb optical properties (e.g., fluorescence, strong UV-Vis light absorption), biocompatibility, non-toxicity and facile synthesis procedure [22,23,24]. Moreover, due to their rich surface functionality, CDs present not only excellent water solubility and enhanced interactions with other molecules or materials, but also oxidant and antioxidant activity, which render them one of the most attractive candidates as nanostructured antivirals [25].

In another approach, noble metal NPs, including gold (Au), silver (Ag) or platinum (Pt), are widely employed to improve the photocatalytic performance of semiconductor materials, since they function as irreversible electron traps promoting the efficiency of electron–hole pair separation. Among them, Ag NPs stand out considering their relatively low cost, along with their efficient photocatalytic activity [26,27]. More specifically, Ag NPs exhibit the surface plasmon resonance effect (SPR), which broadens the optical response of the photocatalysts toward longer wavelengths. Additionally, Ag NPs possess a variety of chemically active sites, further promoting the conduction of chemical reactions [26]. The antiviral activity of Ag NPs against a wide range of virus families has been reported by numerous studies, which have highlighted their noteworthy preventive effects, primarily through direct virucidal action or by interfering with virus binding to host cells [28]. Consequently, the combination of TiO_2_ semiconductor with CDs and Ag NPs synergistically enhances photocatalytic efficiency and provides both antiviral and chemical reactivity, resulting into a promising multifunctional hybrid material. To date, some studies have primarily explored the application of TiO_2_/CDs/Ag ternary hybrid systems in the context of photocatalysis, including organic pollutant degradation [26,29], hydrogen production [30] and photocathodic protection [31]. These studies clearly demonstrate the synergistic effect of CDs and Ag NPs in improving charge separation and photocatalytic performance of TiO_2_. However, the applicability of TiO_2_/CDs/Ag hybrid materials in both liquid and gaseous photocatalysis, and importantly for antiviral purposes, remains an unexplored field.

In this study, a multifunctional TiO_2_/N-CDs/Ag heterostructure was developed based on a facile three-step process aiming to combine improved photocatalytic and antiviral performance within a single platform. The main objective of this work was to investigate the role of N-CDs and Ag in the photocatalytic degradation of liquid and gaseous pollutants, along with the antiviral activity against SARS-CoV-2. To the best of our knowledge, this is the first study that presents a TiO_2_/CDs/Ag hybrid material with dual photocatalytic and antiviral activity.

## 2. Materials and Methods

Briefly, N-doped CDs (N-CDs) were firstly produced via hydrothermal treatment of small organic molecules, whereas a TiO_2_/N-CD hybrid material was prepared based on a solvothermal procedure. Following this, a simple thermal process was employed for TiO_2_/N-CD decoration with Ag NPs. The as-prepared materials were studied in terms of their structural and physicochemical properties via X-Ray Diffraction (XRD) Analysis, Micro-Raman Spectroscopy, Fourier Transform Infrared (FT-IR) Spectroscopy, Ultraviolet–Visible (UV-Vis) Spectroscopy and Transmission Electron Microscopy (TEM). The photocatalytic performance of TiO_2_, TiO_2_/N-CDs and TiO_2_/N-CDs/Ag was assessed for the degradation of both liquid (methylene blue, MB) and gaseous (nitrogen oxides, ΝO_x_) pollutants. Additionally, the antiviral activity of the nanomaterials was investigated against SARS-CoV-2, further demonstrating the simultaneous applicability of the hybrid system in environmental remediation and pathogen inactivation. Given its promising photocatalytic and antiviral properties, TiO_2_/CDs/Ag was further examined by incorporating it into a cellulose fiber-based coating, aiming to investigate its potential for protecting everyday surfaces against viruses and contaminants. To the best of our knowledge, this is the first study that presents a TiO_2_/CDs/Ag hybrid material with dual photocatalytic and antiviral activity.

### 2.1. Materials

Titanium dioxide (Mn-doped TiO_2_) was obtained from PCN Materials (PCN Materials, Greece), produced based on the method described by Karafas et al. [32]. Citric acid (C_6_H_8_O_7_, 99.5%) and urea (CH_4_N_2_O, 98%) were obtained from Sigma-Aldrich (Sigma-Aldrich, Steinheim, Germany). Silver nitrate (AgNO_3_) and ammonia (NH_3_, 25%) were purchased from Fisher Scientific (Fisher Scientific, Waltham, MA, USA) and Panreac ITW Reagents (Panreac ITW Reagents, Castellar del Vallès, Spain), respectively, whereas ethanol (99.8%) and methylene blue (C_16_H_18_ClN_3_S) powder were acquired from Thermo Fisher Scientific (Thermo Fisher Scientific, Waltham, MA, USA). Micro-fibrillated Cellulose (MFC) slurry (95–100% *w*/*w*) was obtained from FiberLean (FiberLean, Trethurgy, UK) and filter papers were purchased from Macherey-Nagel (Macherey-Nagel GmbH & Co, Düren, Germany). Vero cells ATCC with identification number CCL-81 were utilized. Dulbecco’s modification of Eagle’s medium (DMEM), fetal bovine serum (FBS), non-essential amino acids, penicillin, streptomycin, L-glutamine, Alexa Fluor^TM^633 and nuclear counter staining (Hoechst 33342) were purchased from Thermo Fisher Scientific (Thermo Fisher Scientific, Waltham, MA, USA). Methanol and phosphate buffer saline (PBS) were obtained from Scientific Laboratory Supplies (Scientific Laboratory Supplies Ltd., Nottingham, UK) and OXOID (OXOID Ltd., Basingstoke, UK), respectively. The absolute antibody was obtained from Absolute Biotech (Absolute Biotech, Lazenby, UK).

### 2.2. Methods

XRD analysis was carried out on a Bruker D8 Advance diffractometer with Cu-K_α_ radiation (Bruker, Billerica, MA, USA). Data were collected over the angular range of 20–75° with a step of 0.02° in the detector position. Micro-Raman spectra were recorded using a Renishaw inVia Raman microscope equipped with a 785 nm laser (Renishaw, Wotton-under-Edge, UK). FT-IR spectra were obtained with a Jasco FT-IR 4200 spectrophotometer (Jasco, Hachioji, Japan) in the range of 400–4000 cm^−1^ and resolution of 4 cm^−1^, using KBr pellets. TEM analysis was performed via a JEM 2100 HR microscope (JEOL Ltd, Tokyo, Japan) equipped with a JED-2300T energy-dispersive X-ray spectrometer and a digital scanning image observation device (STEM, JEOL EM 24511 SIOD, Tokyo, Japan). The optical band gap energy (E_g_) of the samples was measured using a Perkin Elmer Lambda 950 UV/Vis/NIR spectrophotometer (PerkinElmer, Waltham, MA, USA) in the range of λ = 250–2500 nm. The photocatalytic activity of the samples towards MB dye was evaluated via a Varian Cary 50 UV-Vis spectrophotometer (Agilent Technologies, Santa Clara, CA, USA).

### 2.3. Synthesis of N-Doped Carbon Dots (N-CDs)

**Figure 1:**
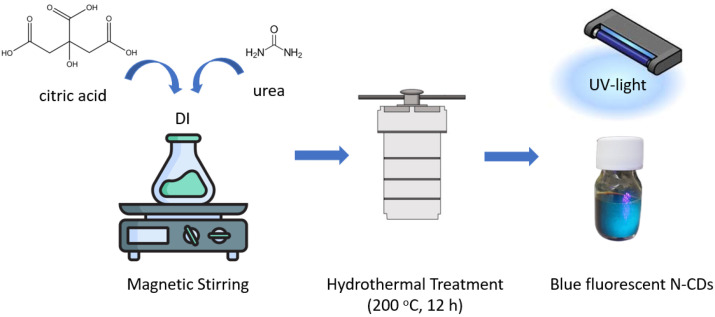
Schematic representation of N-CD synthesis via hydrothermal treatment.

N-CDs were prepared through a facile hydrothermal method, following the procedure described in our previous study [33], using citric acid as a carbon source and urea as both carbon and nitrogen sources. More specifically, citric acid and urea (in a mass ratio of 1:1) were dissolved into DI water, under vigorous magnetic stirring. The colorless aqueous solution was then transferred into a Teflon-lined stainless-steel autoclave and heated at 200 °C for 12 h. The resulting dark brown mixture was centrifuged at 6000 rpm for 30 min and the final Ν-CDs aqueous solution was stored for further utilization. The synthesis procedure of N-CDs is illustrated in Figure 1.

### 2.4. Synthesis of TiO_2_/N-CDs

**Figure 2:**
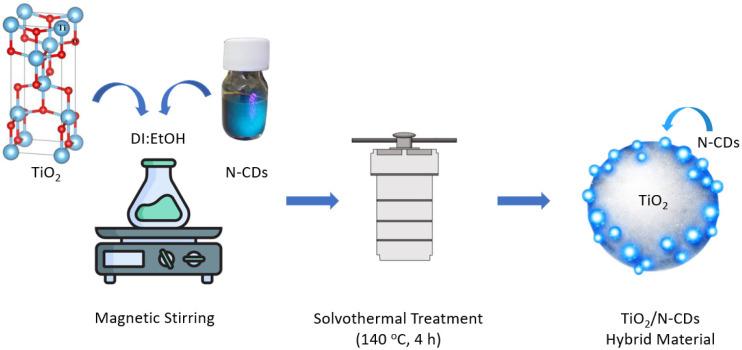
Schematic representation of the solvothermal synthesis of TiO_2_/N-CD hybrid material.

TiO_2_/N-CD hybrid material was synthesized via a simple solvothermal method. Briefly, 0.40 g of TiO_2_ was dispersed into a mixture of DI water and ethanol, followed by the addition of 50.0 μL of CD aqueous solution under vigorous stirring for 4 h. Following this, the mixture was transferred into a Teflon-lined stainless-steel autoclave and heated at 140 °C for 4 h. After the reaction was complete, the mixture was centrifuged at 6000 rpm for 30 min and the precipitate was washed with excess DI water and ethanol and dried at 60 °C, overnight. The synthesis process of TiO_2_/N-CD hybrid material is presented in Figure 2.

### 2.5. Synthesis of TiO_2_/Ν-CDs/Ag

**Figure 3:**
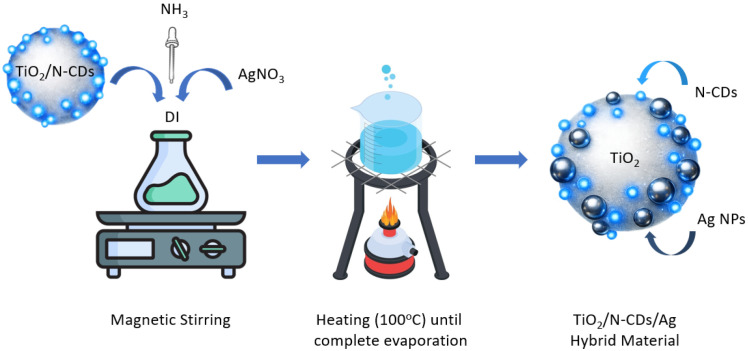
Schematic representation of TiO_2_/N-CDs/Ag hybrid material formation.

Herein, the TiO_2_/Ν-CDs/Ag hybrid material was prepared through a simple thermal process under ambient conditions (Figure 3). More specifically, 0.10 g of the as-synthesized TiO_2_/Ν-CD heterostructure was dispersed into 100.0 mL of DI water, under vigorous magnetic stirring for 30 min. Following this, 0.02 g of AgNO_3_ precursor material was added into the above mixture and the pH was fixed at 12 by the dropwise addition of NH_3_, 25%. Afterwards, the mixture was heated at 100 °C until complete evaporation and a gray precipitate was formed, which was then thoroughly washed with excess DI water and EtOH and dried at 60 °C, overnight.

Moreover, the as-prepared TiO_2_/Ν-CDs/Ag was further incorporated into a cellulose fiber coating for further antiviral testing. Briefly, 25.0 mL of MFC was dispersed into 100.0 mL of DI water, followed by the addition of 0.05 g of TiO_2_/Ν-CDs/Ag hybrid material, under magnetic stirring for 30 min. Subsequently, 8 filter papers were simultaneously dip-coated into the above mixture. Thereafter, the filter papers were sequentially removed from the coating suspension after 0.5 h, 1 h, 2 h, 4 h, 6 h, 8 h, 24 h, and 48 h, respectively, and dried under ambient conditions for future testing.

### 2.6. Photocatalytic Experiments

#### 2.6.1. Liquid Photocatalysis

The photocatalytic performance of the as-prepared samples (TiO_2_, TiO_2_/N-CDs, TiO_2_/N-CDs/Ag) in liquid phase was evaluated by monitoring the degradation of MB dye under natural solar light irradiation. More specifically, a stock aqueous solution of MB with a concentration of 10.0 mg/L was prepared. Following this, 50.0 mg of each powder sample was added into 100.0 mL of the MB initial solution and remained under dark for 60 min, until adsorption equilibrium was achieved. Then, the samples were exposed to natural solar light and monitored over time (15 min, 30 min, 60 min, 120 min, 180 min) via UV-Vis spectroscopy, by measuring the absorbance value of the MB dye aqueous solution at λ_max_ = 664 nm. Herein, it is important to note that the experiments were conducted simultaneously in order to ensure identical natural light conditions and enable reliable comparison of the results. The photocatalytic experiments under natural sunlight were conducted on an open rooftop platform exposed from 10:00 a.m. to 1:00 p.m. in May. Cyclability experiments were also conducted for four consecutive cycles, with each cycle involving 1 h of solar light irradiation, and the results are presented in Figure S1. Furthermore, the stability of the photocatalysts after the last cycle was confirmed by XRD and FT-IR characterization and the results are shown in Figure S2.

#### 2.6.2. Gaseous Photocatalysis

To evaluate the gaseous photocatalytic performance of TiO_2_, TiO_2_/N-CDs and TiO_2_/N-CDs/Ag samples, the NOx degradation was measured in a custom-made photocatalytic chamber. Briefly, the experimental set-up consists of a transparent chamber, where the reaction takes place, with an inlet and outlet gas system, settled in a fully illuminated box. Illumination was provided either by ten fluorescent black light-blue (15 W Philips TLD, Philips, Amsterdam, The Netherlands) lamps or by ten visible common lamps (15 W OSRAM LUMILUX cool daylight, OSRAM, Munich, Germany) positioned in equal distance from the reaction chamber. Initially, the chamber was filled with a specific concentration of NO (~500 ppb). The concentration of NO_x_ and consequently the degradation levels were monitored with a Chemiluminescence NO-NO_2_-NO_x_ Analyzer (Model 42i, Thermo Fisher Scientific, Waltham, MA, USA) connected with the outlet of the inner box.

### 2.7. Antiviral Activity Testing

In the present study, the SARS-CoV-2 omicron BA.2 variant was used as the model for the antiviral application of the nanoparticles. The African green monkey kidney cell line, Vero cells (ATCC: CCL-81), were used as the host cell for the virus. The antiviral activity was assessed by the ability of nanomaterials to inhibit viral infection in relation to the control, without nanomaterials. Nanomaterials were prepared in various concentrations—5, 4, 2, 1, 0.5, and 0 (medium-only control) g/L (*v*/*v*)—in the cell culture medium DMEM with additional 10% FBS, 1% non-essential amino acids, 100 units/mL penicillin, 100 units/mL streptomycin and 1% L-glutamine. The infectious SARS-CoV-2 virus (Omicron BA.2 variant) was prepared at the final multiplicity of infection (MOI) of 0.05 and an equal volume to the prepared nanoparticles was added. The mixtures were incubated either under light irradiation inside a microbiological safety cabinet (CAS model: BioMAT 1, illumination intensity: 900 lux) or under dark conditions (covered with aluminum foil) for 60 min at ambient conditions. The mixture was then transferred to a 96-well plate containing the Vero cell line and incubated for another 18 ± 2 h at 37 °C, 5% CO_2_. The 96-well plate was then fixed with ice-cold methanol, followed by washing one time with 1× PBS. The viral infection will be detected using indirect immunofluorescence assay (IIFA). To evaluate the antiviral properties of coated materials, ten 1 μL droplets of SARS-CoV-2 suspension (titter of 1 × 10^6^ FFU/mL) were deposited onto the coated substrate with or without TiO_2_/Ν-CDs/Ag. The droplets were allowed to remain in contact with the surface for minutes, at ambient temperature. To recover infectious viral particles, 120 μL of culture medium was applied to the surface and pipetted up and down five times over the area containing the deposited droplets then transferred to the 96-well plate containing Vero cells for 18 ± 2 h at 37 °C, 5% CO_2_. The fixation method was applied in the same protocol as mentioned above.

### 2.8. Indirect Immunofluorescence Assay

SARS-CoV-2 infection in Vero cells was detected using an indirect immunofluorescence assay (IIFA) with a monoclonal antibody specific to the viral nucleocapsid protein. Briefly, the primary antibody (anti-nucleocapsid protein, Ab01690-3.0; Absolute Antibody, Oxford, UK) was applied to a 96-well plate containing SARS-CoV-2-infected or uninfected cells treated with TiO_2_, TiO_2_/N-CDs and TiO_2_/N-CDs/Ag samples. Wells containing virus-only and mock-infected cells served as positive and negative staining controls, respectively. Following a 1 h incubation, the antibody solution was removed and PBS was added to wash the unbound antibody. Then, the secondary antibody (Goat anti-Mouse IgG (H+L) Cross-Adsorbed Secondary Antibody), Alexa Fluor^TM^ 633, was added and incubated for another 1 h. Simultaneously, nuclear counter staining was added for cell number quantification. To remove the excessive fluorophores, the plate was washed twice with PBS before the quantification. High-content fluorescence microscopy (CX5) was employed to quantify infection by analyzing nine fields of view per well. Antiviral activity was expressed as the percentage of inhibition relative to the medium-only control. The half-maximal inhibitory concentration (IC_50_) was calculated for each nanomaterial to compare antiviral efficacy.

## 3. Results and Discussion

### 3.1. X-Ray Diffraction (XRD) Analysis

**Figure 4:**
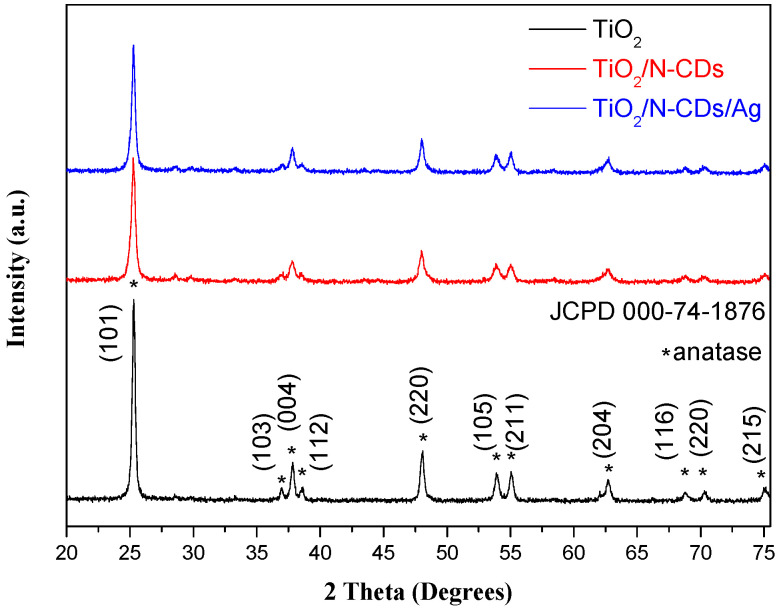
XRD spectra of TiO_2_, TiO_2_/Ν-CDs and TiO_2_/Ν-CDs/Ag.

The crystallographic structure of pristine TiO_2_, as well as TiO_2_/Ν-CDs and TiO_2_/Ν-CDs/Ag hybrid materials, was studied by powder XRD analysis and the results are illustrated in Figure 4. More specifically, the observed diffraction peaks at 2θ = 25.28°, 36.94°, 37.85°, 38.60°, 48.00°, 53.88°, 55.05°, 62.70°, 68.80°, 70.30° and 75.01° are assigned to the (101), (103), (004), (112), (220), (105), (211), (204), (116), (220) and (215) crystal planes of anatase TiO_2_, respectively [34,35]. The aforementioned peaks are also observed in the XRD spectra of both TiO_2_/Ν-CDs and TiO_2_/Ν-CDs/Ag hybrid materials. Noticeably, no diffraction peaks that are correlated with either N-CDs or Ag NPs are observed; this may be attributed not only to their extremely small size and/or their homogeneous distribution onto the TiO_2_ surface, but also to their low content in the final hybrid material. In addition, the crystallite size of TiO_2_ across all samples was estimated according to the Debye–Scherrer equation:

(1) $$d_{hkl}=\frac{0.9\;\lambda }{Β\cos\theta \;}$$

where *d_hkl_* is the average crystallite size, *λ* is the wavelength of the X-rays, B represents the full width at half maximum of the (*hkl*) diffraction peak and *θ* is the Bragg angle corresponding to (*hkl*) reflection. Based on the characteristic (101) diffraction peak of anatase phase, the average crystallite sizes of TiO_2_ were found to be 26.67 nm, 18.71 nm, and 20.94 nm for the TiO_2_, TiO_2_/Ν-CDs and TiO_2_/Ν-CDs/Ag samples, respectively. The obtained results revealed that N-CD integration decreases the average crystallite size of TiO_2_. This could be attributed to the increased lattice strain and defect formation at the nanomaterial interface. Further modification of TiO_2_/Ν-CDs with Ag NPs resulted in a slight increment of the average crystallite size, suggesting a structural reorganization at the ternary interface during the in situ process.

### 3.2. Micro-Raman Analysis

**Figure 5:**
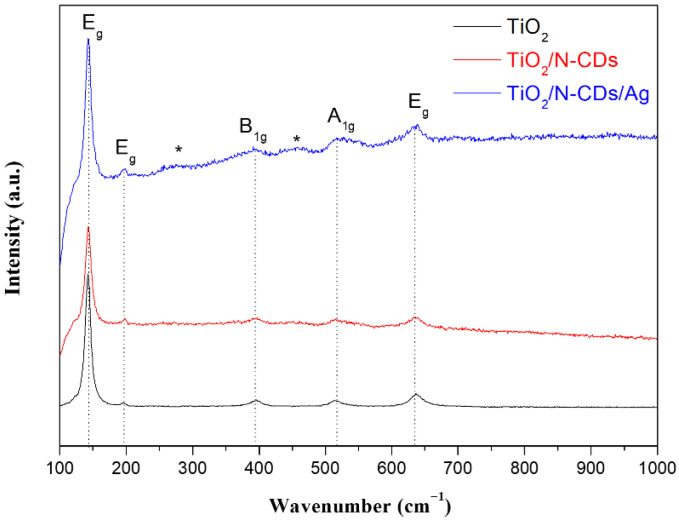
Micro-Raman spectra of TiO_2_, TiO_2_/N-CDs and TiO_2_/N-CDs/Ag. The asterisk (*) indicates the anatase-related Raman peaks revealed by SERS enhancement after Ag NPs decoration.

The structural properties of TiO_2_, TiO_2_/N-CD and TiO_2_/Ν-CDs/Ag samples were also studied via Micro-Raman Spectroscopy and the results are presented in Figure 5. As it is observed in the micro-Raman spectrum of pristine TiO_2_, the peaks located at 141 cm^−1^, 195 cm^−1^, 395 cm^−1^, 513 cm^−1^ and 636 cm^−1^ are assigned to the E_g_ (1), E_g_ (2), B_1g_, (A_1g_ + B_1g_) and E_g_ (3) modes of phonon vibrations in the structure of anatase TiO_2_, respectively [36]. The above peaks are also noticed in both micro-Raman spectra of TiO_2_/N-CDs and TiO_2_/N-CDs/Ag. As shown in Figure S3, no signal related to the characteristic G and D peaks of Ν-CDs (∼1.350 cm^−1^ and ∼1.580 cm^−1^), corresponding to the sp^2^ and sp^3^ carbon hybridization, was identified, which could be attributed to the strong fluorescence of N-CDs. In addition, the intensity of characteristic peaks of TiO_2_ is increased after Ag NP loading. Although Ag NPs are not Raman-active [37], they are known for surface-enhanced Raman scattering (SERS), a phenomenon where the Raman signal of molecules is greatly amplified when they are combined with Ag or Au [38,39]. Furthermore, after Ag NP decoration, two additional Raman peaks located at approximately 270 cm^−1^ and 450 cm^−1^ become visible due to SERS enhancement; these peaks are assigned to anatase phase, in agreement with XRD results.

### 3.3. Fourier Transform Infrared (FT-IR) Spectroscopy

**Figure 6:**
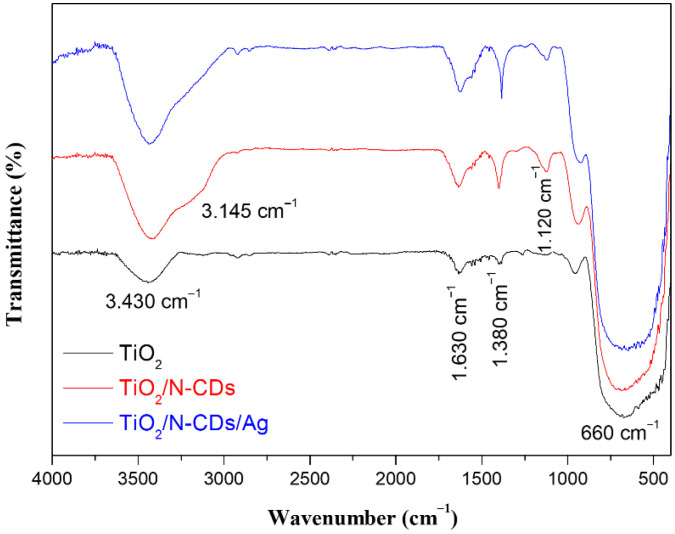
FT-IR spectra of TiO_2_, TiO_2_/N-CDs and TiO_2_/N-CDs/Ag.

FT-IR spectroscopy was employed in order to identify the presence of functional groups and chemical bonds in the as-synthesized materials and the results are presented in Figure 6. In all cases, the characteristic broad peaks located at ∼3.430 cm^−1^ and 660 cm^−1^ are assigned to the O-H of adsorbed water molecules and Ti-O-Ti stretching vibrations, respectively [40]. As it is observed, upon modification of pristine TiO_2_ with N-CDs, the intensity of the absorption peak corresponding to -OH groups is significantly increased. This enhancement is assigned to the presence of abundant surface functional groups introduced by the N-CDs. Furthermore, the peak at 1.630 cm^−1^ is attributed to the bending vibration of O-H, as well as to the C=C stretching vibrations of N-CDs, both of which absorb electromagnetic radiation in the same region [41]. Furthermore, the absorption peaks at 3.145 cm^−1^ and 1.120 cm^−1^ are assigned to the N-H and C-O stretching vibrations, respectively [42,43]. In addition, the absorption peak observed at 1.380 cm^−1^ is related to Ti-O modes [44]. Finally, Ag NPs do not produce an IR absorption signal since they do not present dipole moment changes during vibration [45]; therefore, further investigation (e.g., EDS) is deemed necessary for a comprehensive understanding of the material composition and bonding characteristics.

### 3.4. Transmission Electron Microscopy (TEM)

**Figure 7:**
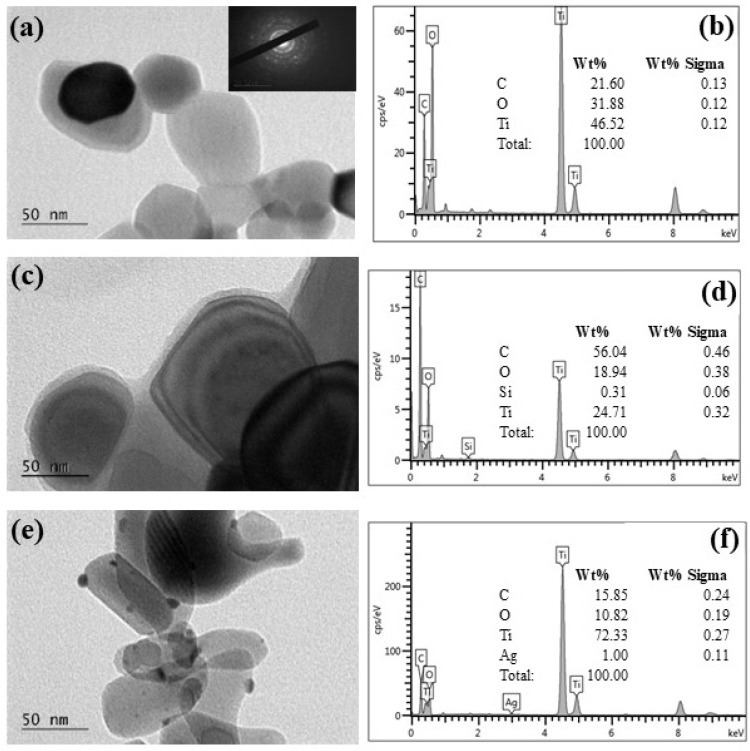
TEM images and EDS analysis of (**a**,**b**) TiO_2_, (**c**,**d**) TiO_2_/N-CDs and (**e**,**f**) TiO_2_/N-CDs/Ag hybrid materials.

The morphological characteristics of TiO_2_, TiO_2_/N-CDs and TiO_2_/N-CDs/Ag samples were studied via TEM and the results are presented in Figure 7. As it is observed in Figure 7a, pristine TiO_2_ NPs exhibit a spherical or semispherical shape, whereas in Figure 7b the presence of Ti and O was verified through EDS analysis; the detected C signal is attributed to the substrate. Notably, Figure 7c reveals that the solvothermal treatment leads to the formation of an approximately 5 nm layer of N-CDs onto the surface of TiO_2_, indicating the successful synthesis of TiO_2_/N-CD hybrid material. Consistent EDS results are observed for the TiO_2_/N-CDs sample (Figure 7d), as the presence of C originated from both the hybrid material and the substrate. Furthermore, the decoration of TiO_2_/N-CDs with Ag NPs was also confirmed, as shown in Figure 7e. As it is observed, Ag NPs with an average size of 15 nm have been successfully formed onto the surface of TiO_2_/N-CDs, whereas EDS analysis of the TiO_2_/N-CDs/Ag sample (Figure 7f) verifies their presence.

### 3.5. Ultraviolet–Visible–Near-Infrared (UV-Vis-NIR) Spectroscopy

**Figure 8:**
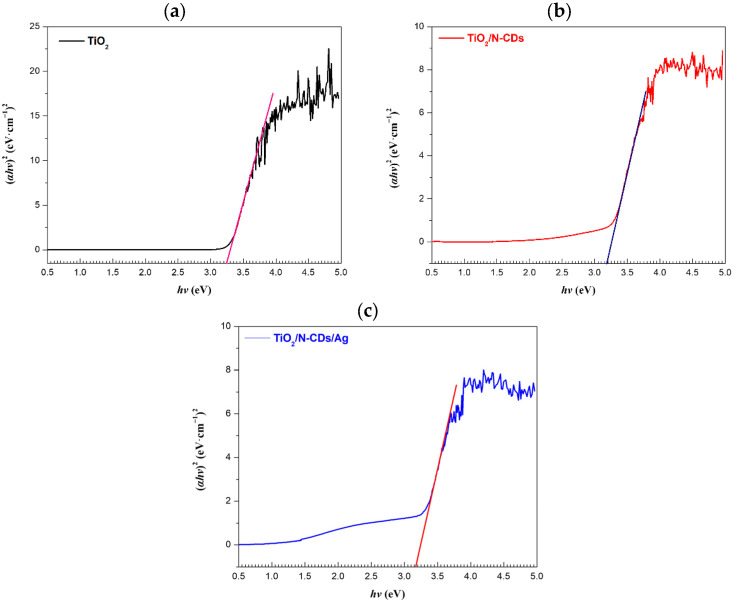
Tauc plots of (**a**) TiO_2_, (**b**) TiO_2_/N-CDs and (**c**) TiO_2_/N-CDs/Ag for the estimation of optical band gap energy (E_g_).

The optoelectronic properties of the samples were investigated via UV-Vis-NIR spectroscopy. More specifically, the optical band gap energy (E_g_) values were calculated using the Kubelka–Munk function

(2) $$F(R)=\frac{{\left(1-R\right)}^{2}}{2R}$$

where *R* is the reflectance of the sample, as well as the Tauc equation:

(3) $$\left(\alpha hv{)}^{n}\;=\;A\;(hv-E_{g}\right)$$

where *α* is the absorption coefficient of the semiconductor, *h* is the Plank constant, *v* is the frequency and A is the material constant [46]. Herein, the value of n = 2 was used for the direct allowed transition, and the E_g_ was determined by generating the Tauc plot, as shown in Figure 8. The values were obtained by extrapolating the linear regime of the curve from the point where the extrapolated line intersects the energy axis. Based on the calculations, the E_g_ of TiO_2_, TiO_2_/N-CDs and TiO_2_/N-CDs/Ag is 3.24 eV, 3.18 eV and 3.16 eV, respectively. The optical band gap of the TiO_2_ semiconductor (∼3.2 eV) is in good agreement with reported values for anatase [47,48]. Upon incorporation of N-CDs, the estimated E_g_ is decreased, suggesting the improvement of visible-light harvesting capability. Further modification with Ag NPs leads to an even more pronounced enhancement of optical absorption in the visible region, along with a slight reduction in the E_g_. Thus, the TiO_2_/N-CDs/Ag hybrid material is considered a promising candidate for visible-light-driven optoelectronic and photocatalytic applications.

### 3.6. Photocatalytic Activity

#### 3.6.1. Degradation of MB

**Figure 9:**
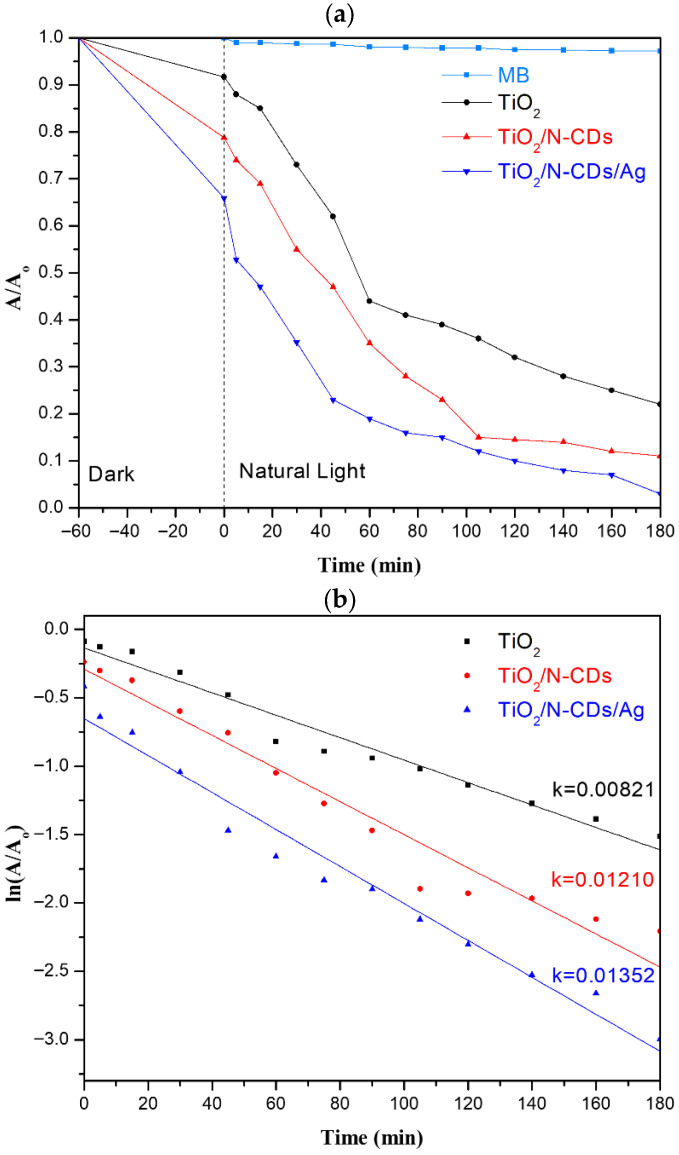
(**a**) Photolysis and photocatalytic degradation of MB dye under natural solar illumination using TiO_2_, TiO_2_/N-CDs and TiO_2_/N-CDs/Ag. (**b**) Kinetic analysis of the degradation process with the corresponding rate constants (*k*) indicated for each sample.

The photocatalytic performance of the samples was evaluated through the degradation of MB, one of the most widely used industrial dyes. In Figure 9a, the results associated with photocatalytic MB reduction as a function of time, under natural solar illumination, are presented. For comparative purposes, MB photolysis is additionally depicted. It is noted that the negative values in the time axis correspond to the period required to establish adsorption equilibrium under dark conditions (Figure S2). To begin with, all samples exhibit a certain degree of MB degradation during the dark period, attributed to the adsorption phenomena taking place onto the photocatalyst surface. In particular, the functionalization of TiO_2_ with N-CDs significantly enhanced its interactions (e.g., hydrogen bonding, etc.) with MB molecules due to the presence of various surface functional groups (-OH, -COOH, -C=O, -NH_2_) of N-CDs [49]. Similarly, further decoration of TiO_2_/N-CDs with Ag NPs led to an additional increase in MB adsorption, as a result of the introduction of new active sites. Upon exposure to natural solar light illumination (t = 0 min), the normalized absorbance decreased for all samples, confirming the activation of the photocatalytic degradation. After 180 min of irradiation, TiO_2_, TiO_2_/N-CD and TiO_2_/N-CDs/Ag photocatalytic materials achieved 78%, 89% and 95% MB degradation efficiency, respectively. As a result, the combination of TiO_2_ with both N-CDs and Ag NPs was proven as the most successful strategy, due to the synergistic effects which improve visible-light absorption and suppress charge carrier recombination. In addition, Langmuir–Hinshelwood kinetic study was used to determine the rate constants, according to the following equation:

(4) $$ln\;\frac{A}{A_{o}}=\;-kt$$

where *k* is the rate constant (min^−1^), *A_o_* is the initial absorbance of the MB, *t* is the photodegradation time (min) and the results are presented in Figure 9b. Based on the kinetic analysis, the calculated k values of TiO_2_, TiO_2_/N-CDs and TiO_2_/N-CDs/Ag against MB are 0.00821 min^−1^, 0.01210 min^−1^ and 0.01352 min^−1^, respectively. Overall, the kinetic results are in excellent agreement with the degradation efficiency and confirm that the combined incorporation of N-CDs and Ag NPs onto the TiO_2_ surface enhances both the reaction rate and the photocatalytic performance.

#### 3.6.2. Degradation of NO_x_

**Figure 10:**
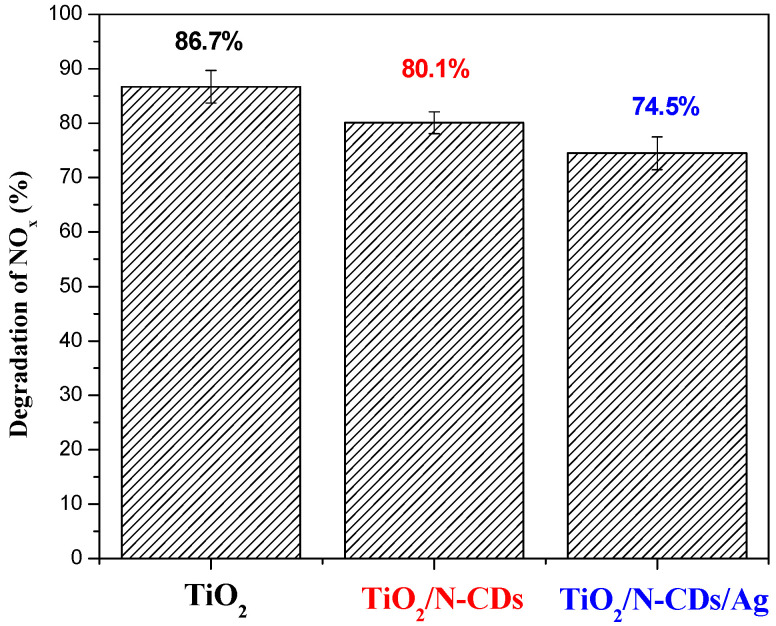
Degradation of NO_x_ under visible-light irradiation over TiO_2_, TiO_2_/N-CDs and TiO_2_/N-CDs/Ag.

Evaluation of gaseous photocatalytic effectiveness of the produced materials took place under the study of NO_x_ pollutants. The initial concentration of the pollutant was chosen with the criterion of reaching similar-level variables in the outdoor environment and according to the ISO: EN 16980-1-202. Moreover, it should be highlighted that all experiments were performed under a dynamic system, meaning that the chamber was continuously supplied with a constant concentration of the tested pollutant during the measurements. Temperature and relative humidity were continuously controlled in the chamber. Figure 10 illustrates that TiO_2_ presents high photocatalytic activity (86.7%) for NO_x_ removal under 5 min of visible-light irradiation, under the experimental conditions adopted in this study. Following, decorating TiO_2_ with N-CDs results in similar photocatalytic efficacy (80.1%) without any remarkable difference in NO_x_ conversion in comparison to core material. The addition of silver shows a relative decrease in efficiency (74.5%) that is possibly attributed to the fact that Ag NPs may promote electron–hole pair recombination, negatively affecting the performance of the hybrid material. Consequently, optimization of the Ag loading might be essential for efficient NO_x_ photocatalytic decomposition [50,51]. Despite the fact that the hybrid materials do not exhibit remarkable enhanced efficacy compared to the core material, their ability to maintain measurable performance in gas-phase photocatalysis remains advantageous.

### 3.7. Antiviral Activity

#### 3.7.1. Antiviral Activity of TiO_2_

**Figure 11:**
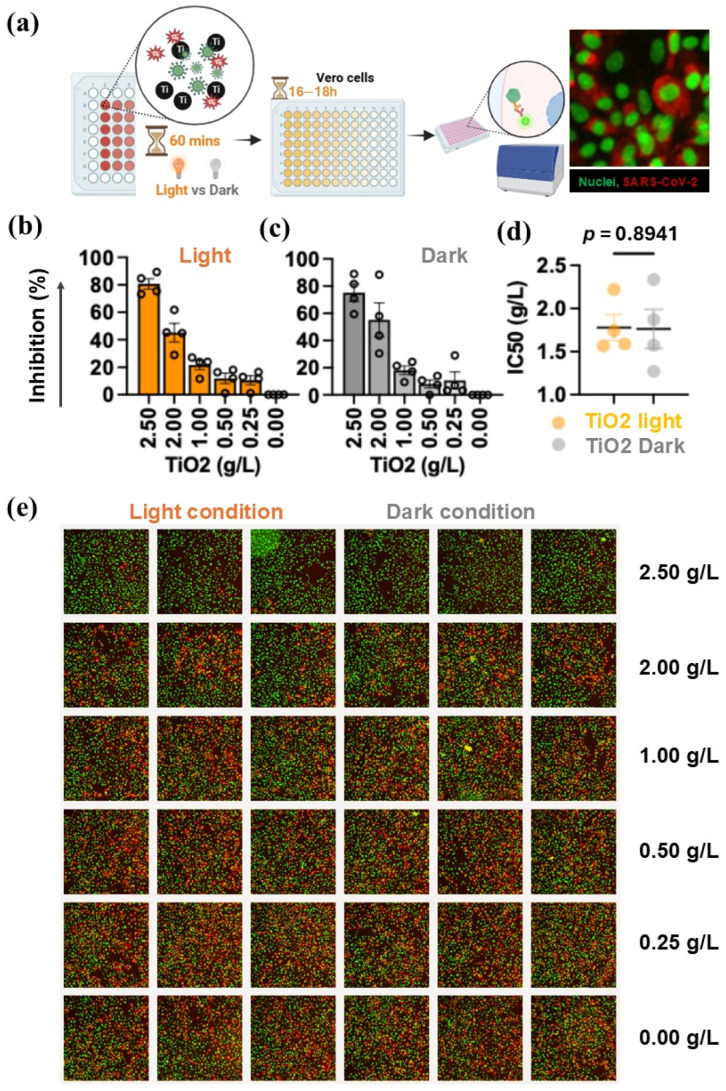
Antiviral activity of TiO_2_ core material. (**a**) The schematic of antiviral test under light and dark conditions. Indirect immunofluorescence test is used to detect SARS-CoV-2 infection. The green dots indicate the nuclei of Vero cells. Infected cells are identified by patchy red color surrounding the nuclei, indicating perinuclear viral protein localization. (**b**,**c**) The percentage of inhibition, under light and dark conditions, respectively. (**d**) Maximal inhibitory concentration (IC_50_). (**e**) Representative images from light and dark conditions. The infected cells are in red color and cell nuclei are green dots. Data represent mean and SD from four independent experiments, and the dots represent data from individual experiments.

The antiviral activity of the TiO_2_ was evaluated against SARS-CoV-2, Omicron BA.2 variant. As shown in Figure 11a, serial dilutions of TiO_2_ were prepared and used to treat viral particles either under light or dark conditions, before inoculation onto Vero cells. The cells were then incubated for 18 ± 2 h to allow viral infection, and infected cells were detected using IIFA. The results revealed that, under light exposure, TiO_2_ inhibited the Omicron BA.2 variant in a dose-dependent manner (Figure 11b,e), consistent with its known photocatalytic properties. At the highest concentration tested (2.5 g/L), TiO_2_ achieved 80.79 ± 7.60% inhibition of viral infection. Interestingly, antiviral activity was also observed when TiO_2_ was kept in the dark during viral treatment (Figure 11c,e), reaching 75.19 ± 12.89% inhibition at the same concentration. This suggests that, although photocatalysis enhances viral inhibition due to oxidation by reactive oxygen species (ROS) (e.g., •OH, O_2_•^−^) [52], TiO_2_ retains a measurable level of antiviral activity independent of light exposure, potentially through mechanisms such as surface charge interactions or adsorption-mediated viral inactivation [53]. However, the IC_50_ values did not differ significantly between light (Mean ± SD, 1.78 ± 0.30) and dark (1.76 ± 0.45) conditions (Figure 11d). Representative images of viral infection are shown in Figure 11e, where the intensity of red fluorescence inversely correlates with the percentage of inhibition relative to the control.

#### 3.7.2. Effects of the TiO_2_ Decorated with N-CDs and Ag NPs on SARS-CoV-2 Inhibition

**Figure 12:**
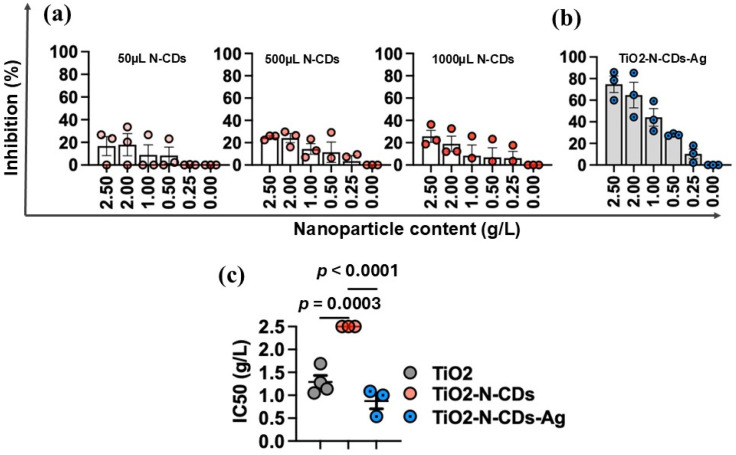
Antiviral activity of TiO_2_ decorated with N-CDs and Ag NPs. Antiviral activity of (**a**) TiO_2_/N-CDs, containing different amounts N-CDs, (**b**) TiO_2_/N-CDs/Ag hybrid material. (**c**) The maximal inhibitory concentration (IC_50_). Data represent mean and SD from at least three biological replicates. The dots represent data from individual experiments.

To evaluate the antiviral activity of the TiO_2_ decorated with N-CDs (TiO_2_/N-CDs) and its Ag decoration form (TiO_2_/N-CDs/Ag), we applied the same experiment approach to all materials. To begin with, TiO_2_/N-CDs samples containing different amounts of N-CDs (50 μL, 500 μL, 1000 μL) were tested in light conditions. The aim was to investigate the effect of N-CD concentration on the antiviral properties of TiO_2_/N-CDs. Based on the results (Figure 12a), similar inhibition of SARS-CoV-2 is observed; this may be attributed to saturation effects, where the active sites or interfacial interactions responsible for viral inactivation are already achieved at low N-CD loading. Furthermore, the antiviral properties of TiO_2_/N-CDs/Ag hybrid material, containing the minimum loading of N-CDs, were also examined and the results are presented in Figure 12b. Notably, the decoration of TiO_2_/N-CDs with Ag NPs resulted in the enhancement of antiviral activity against SARS-CoV-2, Omicron BA.2 variant, compared to both TiO_2_/N-CDs and TiO_2_ samples, under the same experimental conditions. Although standalone Ag nanoparticles were not independently evaluated in the present study, the enhanced antiviral activity of TiO_2_/N-CDs/Ag compared with TiO_2_ and TiO_2_/N-CDs under identical experimental conditions suggests that Ag incorporation contributes positively to the antiviral performance of the hybrid material. In addition, the IC_50_ values of all samples are observed in Figure 12c. More specifically, the lowest IC_50_ values were achieved for TiO_2_/N-CDs/Ag hybrid material (1.24 ± 0.34 g/L (*p* < 0.0001), in comparison with pristine TiO_2_ (1.78 ± 0.30 g/L) and TiO_2_/N-CDs (>2.5 g/L, *p* = 0.0003) with the minimum loading of N-CDs. Consequently, TiO_2_/N-CDs/Ag exhibited the highest antiviral performance, and therefore it was selected for further testing in antiviral coating applications.

#### 3.7.3. Antiviral Activity of TiO_2_/N-CDs/Ag on Paper Coating Substrate

**Figure 13:**
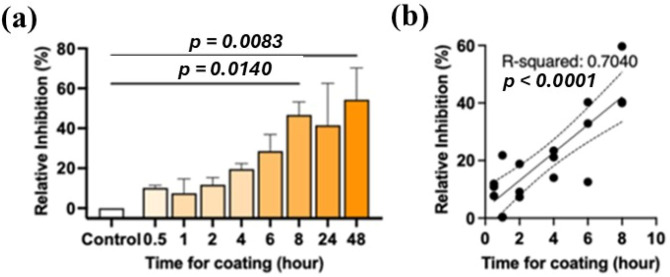
Antiviral activity of TiO_2_/N-CDs/Ag-based MFC application on filter paper substrate. (**a**) Percentage inhibition of SARS-CoV-2 at different coating durations. (**b**) Linear regression analysis showing the correlation between coating time (0.5–8 h) and percentage inhibition.

Based on the antiviral assessments, TiO_2_/N-CDs/Ag hybrid material was selected for further testing as an antiviral component of a cellulose-based coating. For future application of this coating, we firstly performed a preliminary validation using a filter paper substrate to determine the optimal coating duration. As it was mentioned in the experimental part, TiO_2_/N-CDs/Ag combined with MFC was applied onto filter paper, via dip coating, for various time intervals. As shown in Figure 13a, the functional data obtained from assays against SARS-CoV-2 showed that the antiviral activity was improved as the coating duration was increased. Specifically, at early time points (0.5–2 h), the percentage inhibition was low and highly variable, remaining below 20% inhibition. As the duration of dip coating increased, the level of inhibition rose steadily up to approximately 8 h (46.70 ± 11.25%, *p* = 0.0140), after which the antiviral activity reached a plateau. Linear regression analysis across the 0.5–8 h interval demonstrated a significant positive correlation between coating duration and percentage inhibition, indicating that longer coating times were associated with increased antiviral activity (R^2^ = 0.7040, *p* < 0.0001) (Figure 13b). The data suggested that, under the current coating method, a minimum of 8 h was required for TiO_2_/N-CDs/Ag to achieve sufficient deposition on the paper substrate to exert stable and effective antiviral activity.

Notably, SARS-CoV-2 inhibition on filter paper is lower compared to the liquid phase. This may be attributed to the immobilization of the TiO_2_/N-CDs/Ag nanoparticles within the cellulose-based matrix. In the liquid phase, nanoparticles remain well-dispersed and mobile, enabling more effective interactions with the virus. Under these conditions, inhibitory mechanisms may include nanoparticle–virus complex formation and interference with viral attachment, entry, and early-stage replication, since the entire nanoparticle–virus mixture is in direct contact with host cells. Contrary to the liquid phase, the antiviral activity on filter paper is primarily governed by contact-driven virucidal effects. [54] When nanoparticles are deposited onto paper, a significant fraction penetrates into the fiber network. As a result, their accessible surface area is reduced, limiting their interactions with viral particles [55].

Overall, the results demonstrate preliminary evidence for the application of TiO_2_/N-CDs/Ag hybrid materials in antiviral coatings, such as health masks or packaging, while their antiviral performance could be systematically tuned by controlling the coating duration.

## 4. Concluding Remarks

The present study demonstrates that TiO_2_ decoration with N-CDs and Ag NPs results in a multifunctional hybrid material, with enhanced photocatalytic and antiviral properties. More specifically, TiO_2_, TiO_2_/N-CDs and TiO_2_/N-CDs/Ag were utilized for the photocatalytic degradation of water and gaseous pollutants (MB dye and NO_x_, respectively), as well as antiviral inactivation against SARS-CoV-2. Briefly, TiO_2_ was initially modified with N-CDs through a solvothermal method, forming the TiO_2_/N-CD hybrid material, followed by further decoration with Ag NPs, via a facile heating method, under ambient conditions. As it was observed by a series of characterizations (XRD, micro-Raman, FT-IR, TEM and UV-Vis), TiO_2_ was successfully decorated with N-CDs as well as Ag NPs. Based on liquid-phase photocatalytic evaluations, TiO_2_/N-CDs/Ag achieved a degradation efficiency of 95% towards MB dye, which was increased compared to TiO_2_ and TiO_2_/N-CDs; this enhancement highlights the synergistic interactions between N-CDs and Ag NPs on the TiO_2_ surface. In gaseous NO_x_ removal, TiO_2_ itself already exhibited high performance and its modification preserved measurable performance, though Ag loading caused a slight decrease, indicating a tradeoff between liquid- and gas-phase photocatalysis. Antiviral tests against SARS CoV-2 Omicron BA.2 revealed light and dose-dependent inhibition by pristine TiO_2_ with additional dark activity. As it was observed, TiO_2_/N-CDs/Ag presented the lowest IC_50_ value (1.24 ± 0.34 g/L), as opposed to TiO_2_ and TiO_2_/N-CDs, identifying it as the most effective antiviral nanoarchitecture. Furthermore, when incorporated into a cellulose fiber coating, its antiviral activity increased along with the time of dip coating and plateaued around 8 h, supporting the feasibility of TiO_2_/N-CDs/Ag as an active component for the antiviral protection of surfaces, such as packaging. Overall, the study establishes TiO_2_/N CDs/Ag as a promising multifunctional material for addressing both organic pollutants and viral contamination under light conditions.

## Data Availability

The original contributions presented in this study are included in the article/Supplementary Material. Further inquiries can be directed to the corresponding authors.

## Supplementary Materials

The following supporting information can be downloaded at: https://www.mdpi.com/article/10.3390/ma19102084/s1, Figure S1: Recyclability study. Photocatalytic degradation of MB via exposure of TiO_2_, TiO_2_/N-CDs and TiO_2_/N-CDs/Ag in natural light for 1 h, upon five consecutive cycles.; Figure S2: Evaluation of structural stability. XRD and FT-IR spectra of TiO_2_, TiO_2_/N-CDs and TiO_2_/N-CDs/Ag after utilization for four photocatalytic cycles.; Figure S3: Micro-Raman spectra of TiO_2_, TiO_2_/N-CDs and TiO_2_/N-CDs/Ag (wavenumber range: 100-2000 cm^−1^).; Figure S4: Results of dark tests. MB adsorption capacity of TiO_2_, TiO_2_/N-CDs and TiO_2_/N-CDs/Ag at different times. All samples presented an adsorption equilibrium at approximately 60 min.; Figure S5: FT-IR spectra of the as-prepared N-CDs, which were utilized for the ex situ synthesis of the TiO_2_/N-CD hybrid material.
