# Supplementary material for: Carbon Dots-TiO2 Decorated with Ag Nanoparticles for Efficient Photocatalytic and Antiviral Applications
Source: Materials (Basel). 2026 May 15;19(10):2084. doi: 10.3390/ma19102084 (PMC13208284; doi:10.3390/ma19102084)
Supplement: Supplementary file 1 [file materials-19-02084-s001.zip › materials-4300521-supplementary.pdf]

Carbon Dots-TiO_2 Decorated with Ag Nanoparticles for Efficient Photocatalytic and Antiviral Applications

Alexandra Karagianni [1], Adamantia Zourou [1], Aekkachai Tuekprakhon [2], Afroditi Ntziouni [1], Anna-Maria Tavlaridi [1], Ioanna Kitsou [3], Dimitra Katerinopoulou [4], Aspasia Stoumpidi [4], Georgios Kiriakidis [4], Zania Stamataki [2] and Konstantinos V. Kordatos [1]

[1] School of Chemical Engineering, National Technical University of Athens (NTUA), 9 Iroon Polytechniou St., Zografou, 15780 Athens, Greece
[2] Centre of Liver and Gastrointestinal for Research, Department of immunology and Immunotherapy, School of Infection, Inflammation and Immunology, College of Medicine and Health, University of Birmingham, Birmingham B15 2TT, UK
[3] School of Mining & Metallurgical Engineering, National Technical University of Athens, 9 Iroon Polytechniou St., Zografou, 15780 Athens, Greece
[4] Photo-Catalytic Nano Materials (PCN) Materials, Craftsmen Industrial Park of Heraklion, 5 Knossou St., Anopolis, 70008 Heraklion, Greece

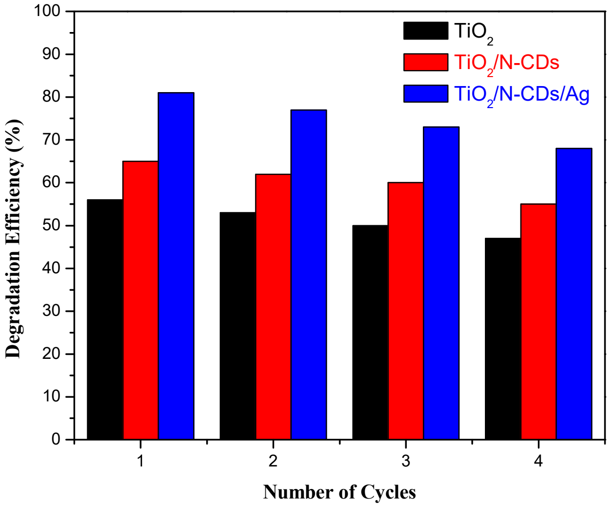

Figure S1. Recyclability Study. Photocatalytic degradation of MB via exposure of TiO_2, TiO_2/N-CDs and TiO_2/N-CDs/Ag in natural light for 1h, upon five consecutive cycles.

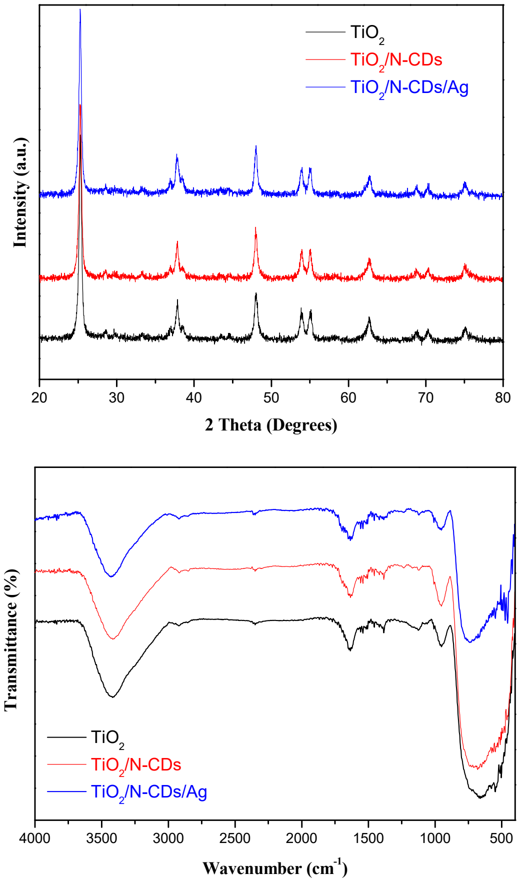

Figure S2. Evaluation of structural stability. XRD and FT-IR spectra of TiO_2, TiO_2/N-CDs and TiO_2/N-CDs/Ag after utilization for four photocatalytic cycles.

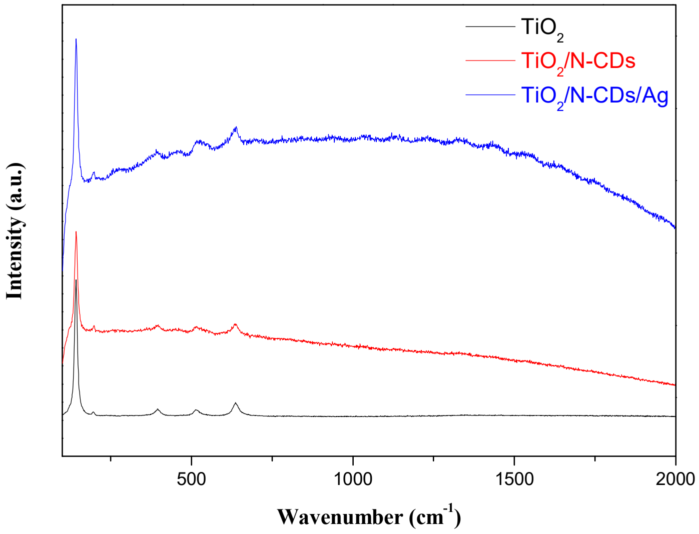

Figure S3. Micro-Raman spectra of TiO_2, TiO_2/N-CDs and TiO_2/N-CDs/Ag (wavenumber range: 100-2.000 cm^{-1}).

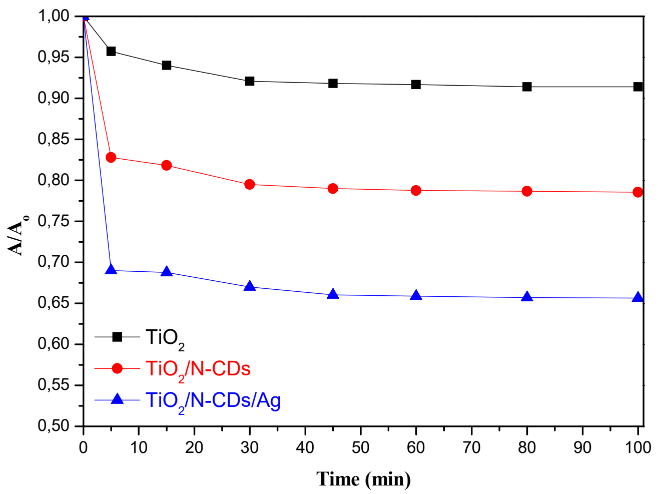

Figure S4. Results of dark tests. MB adsorption capacity of TiO_2, TiO_2/N-CDs and TiO_2/N-CDs/Ag at different times. All samples presented an adsorption equilibrium at approximately 60 min.

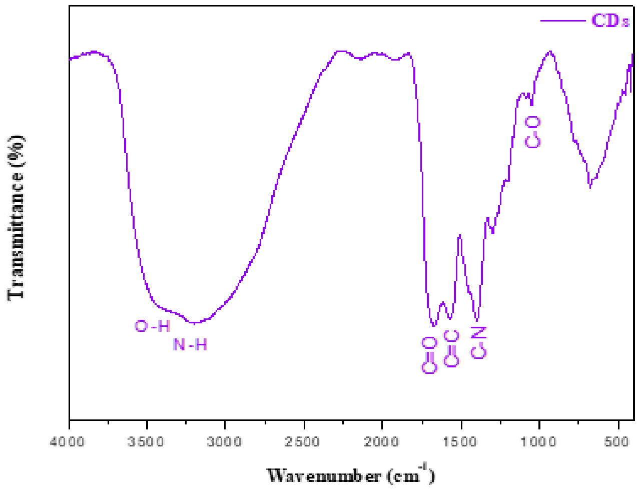

Figure S5. FT-IR spectra of the as-prepared N-CDs, which were utilized for the ex-situ synthesis of the TiO_2/N-CDs hybrid material.
